# An Improved Clustering Algorithm of Tunnel Monitoring Data for Cloud Computing

**DOI:** 10.1155/2014/630986

**Published:** 2014-04-02

**Authors:** Luo Zhong, KunHao Tang, Lin Li, Guang Yang, JingJing Ye

**Affiliations:** ^1^Department of Computer Science and Technology, Wuhan University of Technology, Wuhan 4300702, China; ^2^Department of Computer and Information Science, Hunan Institute of Technology School, Hunan 421002, China; ^3^Collaborative Innovation Center of Hubei Province for Information Technology & Service of Elementary Education, Hubei 430070, China

## Abstract

With the rapid development of urban construction, the number of urban tunnels is increasing and the data they produce become more and more complex. It results in the fact that the traditional clustering algorithm cannot handle the mass data of the tunnel. To solve this problem, an improved parallel clustering algorithm based on *k*-means has been proposed. It is a clustering algorithm using the MapReduce within cloud computing that deals with data. It not only has the advantage of being used to deal with mass data but also is more efficient. Moreover, it is able to compute the average dissimilarity degree of each cluster in order to clean the abnormal data.

## 1. Introduction

At present, with the rapid development of municipal construction in our country, the tunnel of urban is developing progressively. The tunnel data is becoming more and more complex. The clustering algorithm based on partition is of simplicity and accuracy, and it has been widely applied in scientific researches and production practice. As a classical clustering algorithm, *k*-means, which is based on partition, is a hot topic all the time. However, tunnel data has already entered the level of massive amounts of data, taking the time, space, and data volume complexity [1] into account. The traditional clustering algorithm *k*-means has been unable to deal with these data.

According to the characteristics of the *k*-means, to use the parallel computing based on MapReduce in the cloud platform imperative. The probability of cloud computing is proposed by Google; it is a kind of calculation model that combines several technologies; it also has some characteristics, such as high reliability, high extensibility, and super large scale. After that, IBM also brought the cloud computing platform [2] so that clients could be ready to use them. Cloud platform, using Hadoop technology, can realize large-scale distributed computing and processing. It is mainly made up by Hadoop distributed file system and MapReduce programming model [3] which was widely used in cloud computing.

In addition, the MapReduce model is a distributed parallel programming model, which was proposed by Google Labs. It can deal with problems including large datasets with computer cluster, and that makes it become a mainstream of the parallel data processing model of cloud computing platform [4]. The MapReduce model also can easily solve the problems that *K*-means cannot handle, such as processing large data and demanding bigger serial overhead.

The city tunnel monitoring data include a variety of forms; they contain the instantaneous of traffic lane, speed, occupancy rate, wind speed, temperature, humidity, light intensity, and CO concentration [5]. They are multidimensional data from different equipment so that it can ensure the effectiveness of the experimental data [6]. With the continuous development of data processing technology, people begin to analyze huge amounts of data statistics [7] by using various data mining techniques and tools. The purpose of cluster data is to use relevant technology to classify the extracted data from various data sources while finding the abnormal data.

However, the tunnel monitoring system demands real-time higher. At the same time, the collected data storage density is intensive while the data storage is also large [8]. It is such a large amount of data that it cannot use serializing approach to find out the abnormal data. Moreover, it is not able to cluster [9]. Therefore, we use the distributed processing technology to deal with data by cloud computing [10].

## 2. The Introduction of Cloud Platforms

The cloud platform we normally refer to is the platform which can develop easily and deal with processing of mass data in parallel Hadoop [11]. It is mainly composed of the distributed file system, which is called HDFS and the calculation model, which is called MapReduce [12].

### 2.1. Hadoop Distributed File System (HDFS)

**Figure 1:**
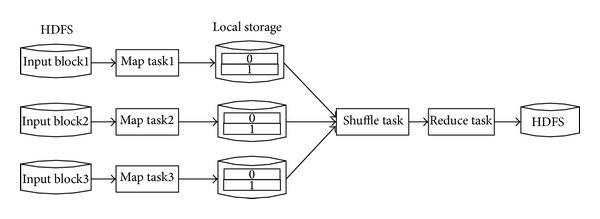
The interaction figure of HDFS and MapReduce.

HDFS has adopted the master/slave structure [13], which is principally composed by Client, Name Node, Secondary, Name Node, and Data Node. Every HDFS cluster consists of a unique management node (the Name Node) and a certain number of data nodes (the Secondary Node), and every node is an ordinary PC. HDFS is very similar to the stand alone; it also can build directory, create, copy, delete files, check the file content [14], and so forth. HDFS is a distributed file system which possesses high fault-tolerant; it can provide high throughput data access [15], which is fairly suitable for the application in large datasets. The interaction process between HDFS and MapReduce is shown in Figure 1.

### 2.2. The Calculation Model of MapReduce

MapReduce is a highly efficient distributed programming model [16] which is developed by Google. It can abstract problems highly and make the problem become simple and it is mainly particularly used for large-scale (TB) data processing [17]. The work process of MapReduce is mainly composed of three parts, Map, Shuffle, and Reduce.

1. The process of Map: Map reads data from the HDFS [18], and the data is divided into several independent shards (Split) and through the Map function iteration parsed into key/value pair 〈key/value〉.
2. The process of Shuffle: Sorting provisional results is generated by the Map phase, according to the key value, dividing the map production temporary data (key/value) into several groups (partition), and, finally, every partition will give a the Reduce Task processing in the form of 〈key, value  list〉.
3. The process of Reduce: reading from the remote node 〈key, value  list〉 and merging them through the reduce function, and in the end, transferring the final result into the HDFS [19].

## 3. Traditional Clustering Algorithm and Its Parallel

### 3.1. Traditional (Serializing) *k*-Means Clustering Algorithm

So far, the commonly used clustering analysis algorithms are composed of the following five categories: the algorithms based on classification, the algorithms based on the hierarchy, the algorithms based on density, the algorithms based on grid, and the algorithms based on model-based [20]. Studies show that tunnel data storage density is large, so algorithms based on data partition are more applicable to tunnel. But *k*-means is a typical representative of algorithms based on partition. *k*-means algorithm; a partition clustering method [21], which regards an average value as the center of the cluster, and the specific steps of serializing *k*-means clustering method are as follows:

1. selecting several objects from the original dataset randomly, and treating them as the initial clustering center,
2. to calculate the distances between the other objects and the center and assign them to the class of the closest center,
3. for each class, working out the means of all the objects as a new center,
4. repeat (2) and (3) until the standard measurement function begins to converge.

### 3.2. The Parallel *k*-Means Clustering Algorithm

Although the sequential *k*-means algorithm is simple and convenient, a lot of defects still exist. The time complexity of *k*-means is *O*(*n*∗*k*∗*t*); in addition, *n* is the number of all the objects, *k* denotes the cluster number of clustering, and *t* represents the number of iterations [22]. Thus it can be seen that the time complexity of sequential algorithm is very high; the iteratively replaced part consumes the most time during this operation, and it is easier for parallel processing [23]. The MapReduce process of parallel *k*-means is as follows.

(*1) The Process of Map*. The Map phase of the MapReduce process is performed by several Map functions. According to the number of input splits, there will be Map function which has some inputs to calculate the distance between every data object and the current cluster center. Furthermore, we can tag the corresponding cluster number for data objects according to the distance. The cluster number is the number of cluster center which is closest to the object. Its input is that all the data objects waiting on clustering and the clustering center during the previous iteration (or the initial center); its input is in the form of (key/value). In key/value pairs, key is corresponding to the offset in current data samples which is relative to the starting point, while value is corresponding to the vector of strings in the current data sample which is composed of each dimension coordinate. After the Map phase, the result will be in the form of key/value pairs to output the clustering cluster number and recorded value.

**Figure 2:**
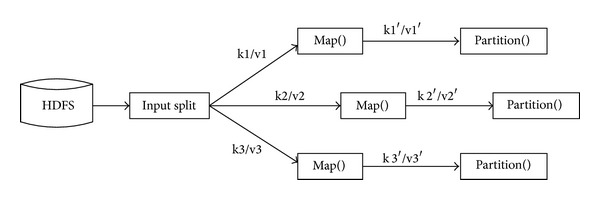
The execution figure of Map.

**Figure 3:**
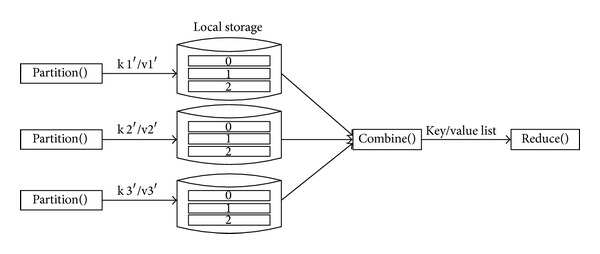
The execution figure of Shuffle.

Its execution process is shown in Figure 2.

(*2) The Process of Shuffle.* There are two main functions, Partition and Combine. Finishing it, the input during this phase is the key/value pair which was worked out during the Map phase, and it is mainly used before entering the Reduce phase, combining the data locally in order to reduce the consumption of data communication in the process of iteration. First of all, the Partition function sorts the provisional result which was produced during the Map phase and divides the temporary data (key/value pairs) which was produced by map into several groups (Partition). The key which was input is corresponding to the cluster number of clustering, while values are corresponding to vector of strings which are composed by dimensional coordinates. Then, the Combine function will combine group and count the number of data samples and the sum of all the data coordinates; in addition, the outputs which were in the form of key/value pair (key/value list) are given to different Reduce functions. Its execution process is shown in Figure 3.

(*3) The Process of Reduce.* Reduce function accepts the key/value pairs (key and value list) which were worked out by Combine function. Among them, corresponding to the key is the number of data samples, and the values list is corresponding to the vector of strings which are composed by dimensional coordinates. By adding string vectors of every dimensional coordinates, and combining with the number of the data, to work out the new cluster center which is input in the form of key/value pairs into the next MapReduce process.

## 4. The Good Center Parallel *K*-Means (GCPK) Algorithm

### 4.1. The Calculation Method of Dissimilarity Degree

This experiment uses Euclidean distance as a measurement standard to calculate the similarity degree among data in the cluster. In this article, the average dissimilarity degree mentioned refers to the average Euclidean distance between the data within the cluster and the cluster center. If the average dissimilarity degree is lower, the data within the cluster will be more similar; in other words, the results of clustering are more ideal. The Euclidean computationalformula is

(1) $$\begin{array}{c} d\left(x,y\right)=\sqrt{\overset{m}{\underset{i=1}{\sum }}{\left(x_{i}-y_{i}\right)}^{2}}. \end{array}$$

The “*m*” denotes the dimension value of the array.

### 4.2. The Defects of Parallel *K*-Means Clustering Algorithm

*K*-means parallel clustering algorithm solves the problem of the iteration time that *k*-means spends very well, but it does not handle the selection problem of *k*-means initial center. In order to choose appropriate initial center, reduce unnecessary iteration, and decrease the operation time and improve efficiency, in view of the tunnel data, we proposed the *K*-means parallel improved algorithm. Good center parallel *k*-means algorithm, whose abbreviation is GCPK algorithm. In the dataset, the algorithm can find out the algorithm firstly find out a point as the center, which can separate data on average basically; in other words, it is appropriate to be the good center for the initial center, and then to parallelize the* k* means clustering. Finally, based on the clustering results, the abnormal data will be analyzed.

### 4.3. The Implementation of GCPK Algorithm

(*1) To Get the Initial Center through Relative Centroid. k*-means clustering method is a typical method based on clustering; in the *k*-means clustering method, because of replacing the center constantly [24], the selection of initial center has a great influence on *k*-means clustering algorithm [25]. In order to decrease the times of replacement, and make the *k*-means clustering become more efficient, it is necessary to find the initial center which is relative to the center and roughly divide the dataset.

Because the tunnel data has the characteristics of nonnegativity, tunnel data can be regarded as multidimensional data graphics, which is composed of *n*-dimensional data. Regarding each data as their own dimensional coordinates, find out the point *Q*1 (*i*_1_, *i*_2_,…, *i*_*n*_) which is closest to the origin of coordinates and the point *Q*2 (*j*_1_, *j*_2_,…, *j*_*n*_) which is farthest away from the origin.

To connect *Q*1 and *Q*2 with a line segment, and divide the line segments into *k* + 1 parts (this *k* here is the same as the *k* in *k*-means), regard each data as a particle, and there are *k* equal diversion points on the line segment (not including the endpoint). Furthermore, the coordinate of the *t* (0 < *t* < *k* + 1, *t* ∈ *Z*) equal diversion point is (*P*_*t*1_, *P*_*t*2_, …, *P*_*tn*_), and *n* denotes the number of dimensions. So *P*_*tm*_ = (*m*/*k*)(*i*_*m*_ + *j*_*m*_), *m* = 1, 2, …, *n*. These *k* points obtained are the points in the center position which can average the multidimensional data graphics, and we regard the *k* points as the initial center points.

(*2) The Specific Implementation of GCPK Parallel Clustering*

1. Upload the dataset to the HDFS.
2. Upload the initial center to the HDFS.
3. According to the original data and the previous iteration (or initial center), calculate the current iteration with the Map function.
4. Calculate the sum of the points' number and weight in the same cluster and recount the clustering center of cluster.
5. Do not control the iterative process until the maximum number of iterations is exceeding or all the clustering has been convergent, at this time, calculate the average dissimilarity degree and the maximum dissimilarity degree within each cluster of all clusters; in addition, count the average dissimilarity degree and the maximum dissimilarity degree in the whole cluster.

(*3) The Analysis of Abnormal Data*. Download the result of clustering to local through HDFS and convert binary serialization format file into a text file. Analyzing the results file, and getting the average dissimilarity degree and the maximum dissimilarity degree in every cluster, counting the radius of every dimension, combining the weight of each dimension data for cleaning abnormal data. Because the amounts of data are huge and the data quantity is too large, it is unable to complete artificial recognition and use the simple method to identify the abnormal data. It is necessary to use cloud computing technology to clean the abnormal clustering results.

(1) Read the average dissimilarity degree *D*_*i*_ in every cluster and the dimension radius *R*_*j*_. Furthermore *i* = 1,2,…, *k*, and *k* are the cluster's numbers, *j* = 1,2,…, *n*, and *n* are the dimensions.
(2) Calculate the weight *Q*_*j*_, which is within the cluster. *Q*_*j*_ = *t*_*j*_/*n*. The “*t*” is the number of times in which the data of dimension *j* come out; the “*n*” is the total dimension.
(3) Work out the reasonable range of dissimilarity degree dis(*i*) in clusters; in addition
  (2) $$\begin{array}{c} \text{dis}\left(i\right)=\sqrt{D{\left(i\right)}^{2}+\overset{n}{\underset{j=1}{\sum }}Q_{j}*R_{j}^{2}} \end{array}$$
  *i* = 1,2,…, *k*, *k* denotes the number of all clusters.
(4) Test the data object which is got from MapReduce according to the data objects. Euclidean distance of the cluster center (dissimilarity degree) and the reasonable range of dissimilarity degree within clusters, and to judge whether the data is abnormal or not. If dissimilarity degrees are beyond reasonable range, we can know it is the abnormal data and record the number.

## 5. Experimental Environment and Results Analysis

### 5.1. Experimental Environment

#### 5.1.1. Single-Machine Environment

This paper uses MATLAB2010 single-machine environment; the operations of the specific environment are the Intel(R) Core(TM) i3 CPU 2.53 GHz, 4 GB of memory, and a 64-bit operating system.

#### 5.1.2. Distributed Environment

In this paper, the experiment of distributed environment uses open source cloud computing platform hadoop, whose version is 1.1.2. Specific cloud cluster is as follows: one machine runs as the Name Node and Job Tracker while the other 19 run as a Catano and Task Tracker. The experimental cloud platform uses the VM technology; specific software configuration is Cream ware workstation-full-9.0.2, a 64-bit Ubuntu-12.04.3, JDK version is jdk1.7.0_21, and eclipse version is Linux version.

#### 5.1.3. Data Source

The data for clustering analysis are from the tunnel monitoring data, including flow, temperature, wind speed, illumination intensity, CO concentration, speed, wind speed, and so on.

### 5.2. The Experimental Data

This experiment has adopted the five datasets which were gathered from tunnel monitoring system of Wuhan Fruit Lake; respectively, they were DataSet1, DataSet2, DataSet3, DataSet4, and DataSet5. Furthermore, the seventh dimensions tunnel data record has been covered 42993 times by the DataSet1, which can be used for single experiment and distributed experiment. The thirtieth dimensions tunnel data record has been covered, respectively, 42775 times, article 65993 times, article 100 000 000 times, and article 2000 000 000 times by DataSet2–DataSet5.

### 5.3. Analysis of Experimental Result

*Experiment* *1*. There are 42993 tunnel data whose dimension value is 7; there are 42993 tunnel data whose dimension value is 7; the ideal value of these data is 5, under the condition of single machine; MATLAB was used to complete *k*-means serial clustering algorithm [26], Clara clustering algorithm [27], and RICA clustering algorithm [28] in the case of the distributed cluster to accomplish *k*-means parallel clustering algorithm with hadoop [29] and to cluster the tunnel data with GCPK; the results are shown in Table 1 under the condition of two kinds of the clustering algorithm.

**Figure 4:**
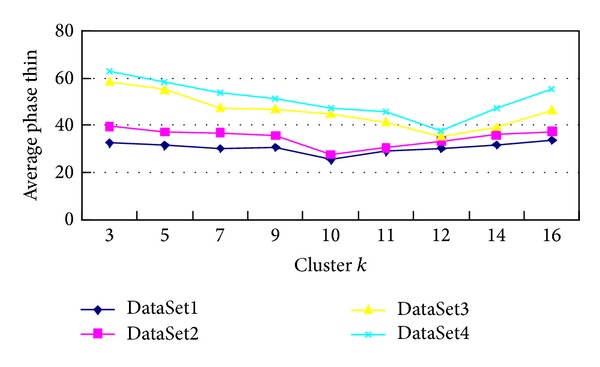
*k* values for experiments.

*Experiment* *2*. For *k*-means clustering, the initial value of* k* has great influence on the clustering results, and the traditional clustering cannot analyze the* k* in the huge amounts of data. This experiment adopted GCPK algorithm, respectively. In terms of four datasets, which, respectively, are DataSet1, DataSet2, DataSet3, and DataSet4, we choose the different* k* values for them to conduct experiments. And the results are shown in Figure 4.

*Experiment* *3*. According to the tunnel monitoring data collected from DataSet2 to DataSet5 (the dimension value is 30), under the distributed circumstances, clustering with *k*-means parallel algorithm and *k*-means parallel improved algorithm severally; the cluster number* k* would find ideal value through GCPK; the time cost in Clustering was shown at Table 2.

## 6. The Conclusion and Expectation

Experimental results show that GCPK, the improved algorithm of *k*-means parallel clustering algorithm, has a better clustering result than the traditional clustering algorithm and has a higher efficiency. It is very convenient to clean the abnormal data for the clustering results, in terms of massive data, such as tunnel data; it has shown its strong practicability. At present, due to the limited data form, we are only able to do preliminary clustering about structured document. However, after the relevant documents for clustering of data protection and the processing of unstructured documents, we will do further researches on the file data protection after clustering and the processing of unstructured documents in the future.

## Figures and Tables

**Table 1:** Thin contrast of DataSet1.

	Serializing *k*-means	Clara	RICA	Parallel *k*-means	GCPK
Cluster (*k*)	5	5	5	5	5
Average phase thin	23.2027	21.8868	19.0924	16.0240	16.0226
Maximum phase thin	105.2276	113.2348	96.5631	78.2311	77.3419
Abnormal finding	83.22%	89.36%	89.53%	93.23%	93.3%

Note: the average phase thin and maximum phase thin are the mean values of each cluster.

**Table 2:** Clustering time.

Dataset	cluster *k*-means	Serializing *k*-means	Parallel *k*-means	GCPK
DataSet2	10	20 min 18 s	7 min 50 s	7 min 22 s
DataSet3	10	27 min 35 s	9 min 21 s	8 min 9 s
DataSet4	12	incalculable	18 min 33 s	16 min 20 s
DataSet5	12	incalculable	29 min 9 s	23 min 15 s
